# Factors influencing smoking cessation attempts and success in Iranian male adults: national survey data

**DOI:** 10.1186/s12889-024-19187-1

**Published:** 2024-06-20

**Authors:** Zohreh Manoochehri, Fatemeh Rajati, Maryam Rezaei, Javad Faradmal

**Affiliations:** 1 Department of Biostatistics, Student Research Committee, Hamadan University of Medical Sciences, Hamadan, Iran; 2 Research Center for Environmental Determinants of Health, Health institute, Department of Health Education and Health Promotion, School of Health, Kermanshah University of Medical Sciences, Kermanshah, Iran; 3 Health System observatory secretariat national Institute for Health research(NIHR), Tehran University of Medical Sciences (TU MS), Tehran, Iran; 4 Modeling of Noncommunicable Diseases Research Center, Department of Biostatistics, School of Public Health, Hamadan University of Medical Sciences, Hamadan, Iran; 5 Department of Biostatistics, School of Public Health, Hamadan University of Medical Sciences, Shahid Fahmideh Boulevard, Hamadan, Iran

**Keywords:** Attempt to quit smoking, Quit smoking, Multinomial regression

## Abstract

**Background:**

Smoking cessation is a dynamic process that often involves a series of unsuccessful quit attempts before long-term abstinence is achieved. To implement interventions that lead to long-term abstinence, it will be necessary to understand the determinants of smoking cessation. Therefore, the main objective of the present study was to determine the effect of factors influencing both smoking cessation attempts and successful smoking cessation in the general population of Iran.

**Methods:**

The data of 1293 participants whose information was obtained through a national cross-sectional study entitled “Survey of Risk Factors of Noncommunicable Diseases in 2016” were analyzed. There were three response levels: “quit attempt and successful quit”, “quit attempt and unsuccessful quit”, and “no quit attempt and unsuccessful quit”. A multinomial logistic regression model was used to assess the effect of covariates on response.

**Results:**

The mean (sd) age of all participants was 47.21 (13.65) years. According to the results, 883 people (68.29%) did not attempt to quit smoking, and of those who attempted to quit smoking, only 64 (15.61%) men were successful. The factors of living in an urban area (OR = 1.71) and past smoking intensity (OR = 1.967) were associated with no quit attempt and unsuccessful quitting. In addition, physician recommendation to quit smoking was a protective factor for no quit attempt and unsuccessful quit (OR = 0.599). Alcohol consumption was also a protective factor against successful quitting for both attempters (OR = 0.351) and nonattempters (OR = 0.359).

**Conclusions:**

Tobacco control programs should be implemented with a greater focus on heavy smokers and alcohol users. In addition, the role of health professionals in encouraging smokers to quit smoking should not be ignored.

## Background

Smoking is known to be a leading cause of preventable death from cancer and respiratory and cardiovascular disease [1]. In addition, smoking threatens mental health by reducing life expectancy, aggression and criminality [2]. Tobacco use imposes high economic costs on health care systems worldwide [3]. According to reports from international sources, in Iran, 2–3 times more than the cost paid for the purchase of cigarettes was spent on medical and health costs related to the treatment of diseases caused by smoking [2]. Although the World Health Organization (WHO) has called for a reduction in tobacco consumption of at least 5.8% [4], the efforts of countries between 1990 and 2015 resulted in a reduction of only 1.6% per year [5]. The daily prevalence of smoking in Iran was reported to be 9.7% in 2016 [6]. According to the WHO report, if the current tobacco consumption situation remains unchanged over the next 40 years, Iran will experience the largest increase in tobacco consumption among countries in the region, indicating that tobacco control policies have not been effectively implemented in Iran [7]. Therefore, there is an urgent need to reduce smoking initiation and promote smoking cessation to combat this growing epidemic [8].

Smoking cessation is a dynamic process that often involves a series of unsuccessful quit attempts before long-term abstinence is achieved [9]. Smoking cessation can be conceptualized as a two-step process, first attempting to quit and then successfully quit smoking [10]. Although a significant number of smokers say they want to quit, only approximately one-third of them try to quit each year, and few of them succeed [11]. Although there are several types of smoking cessation aids on the market, they are rarely used by the general population in low- and middle-income countries due to their high costs, lack of coverage by public insurance plans, and general mistrust of nicotine dependence medications. Therefore, there is a need to explore other feasible interventions [12]. To implement these interventions that lead to long-term smoking cessation, it will be necessary to understand the determinants of smoking cessation. Identifying and focusing on these key factors will help prevent future health care costs imposed by smokers on the nation’s health care system and help improve the level of public health [2].

Previous research has identified factors such as low nicotine dependence, male sex, higher education, being married, consuming fewer cigarettes per day, and having fewer smokers at home as factors associated with cessation [13–16]. However, some studies have produced conflicting results about the role of some of these factors in smoking cessation success. Several studies have concluded that the relationship between socioeconomic status and smoking cessation is complex and that other factors, such as the health care system and smoking cessation behaviors, make this relationship heterogeneous [17, 18]. In addition to socioeconomic factors, although the adverse health effects of smoking are well documented, the relationship between awareness of health problems and smoking cessation success remains unknown [19]. For example, although people with hypertension, chronic heart disease, chronic obstructive pulmonary disease, and diabetes are more likely to quit smoking [19–21], this does not mean that these people have higher quit rates. In terms of the empirical literature, a large number of studies on the determinants of smoking cessation have been conducted in developed and developing countries, which have the following limitations in addition to those mentioned above: (1) most of them have been conducted in specific populations, such as smokers treated in smoking cessation clinics or hospitalized smokers, and fewer studies have been conducted in the general population; and (2) none of these studies have simultaneously examined the factors influencing quit attempts and smoking cessation in the general population of Iran. Therefore, the main objective of the present study was to determine the effect of socioeconomic factors as well as awareness of health status on smoking cessation attempts and successful smoking cessation.

## Methods

This study is a secondary study. It is based on information from the 2016 STEPS smoking questionnaire. The STEPS study, which is a national cross-sectional study, was conducted by the Non-Communicable Diseases Research Center of Iran, and its target population is all adults over 18 years of age. The STEPS smoking questionnaire is a standard questionnaire of the WHO. This questionnaire is self-reported, and its reliability was 80% based on Cronbach’s alpha coefficient. In the STEPS 2016 study, the samples were selected using the multistage cluster sampling method from all provinces of Iran except Qom. The protocol of the STEPS study is published in detail in reference [22].

### Outcome variables and study factors

The population studied in this study is a subset of the STEPS study population, which included all men aged 18 years and older who were either former or current smokers; nonsmokers were excluded from the study. Since tobacco use, is almost negligible among women in Iran, the analysis in this study was conducted using only a male sample. Participants were asked the following questions to determine which of the above groups they belonged to: if subjects answered “yes” to the question “Have you ever smoked?” and “no” to the question “Do you smoke now? “, they were considered to be former smokers. However, if the respondent answered “yes” to both of the above questions, he or she was classified as a current smoker. Otherwise, a person who answered “no” to both questions was considered a nonsmoker and excluded from the study. In this way, the sample size in the present study was 1293 males.

The factors studied were demographic variables, socioeconomic& geographic factors, variables related to smoking behavior, and awareness of health problems and treatment of diseases. The variables of age, place of residence (urban/rural), marital status (married/other: including single, divorced, widowed), years of education (illiterate/1–6 years/7–12 years/>12 years), employment status (employee/worker/self-employed/retired/unemployed/others including student, soldier, unpaid work), monthly household income level (more than $175 vs. $ 175 or less: based on the basic salary of the Ministry of Labor of Iran), and health insurance status (have/do not have) were considered demographic variables and socioeconomic& geographic predictor factors [23].

Variables related to smoking behavior and related questions were as follows: “In the past 12 months, have you been advised by a doctor or health professional to stop smoking? “, “In the past 30 days, has anyone smoked at home or at work? “. Past smoking intensity was also considered a predictive factor. To determine this predictor, participants were asked the question “How many cigarettes have you smoked per day in the past? “. Individuals were classified into three categories: light smokers (less than 10 cigarettes/day), moderate smokers (10–19 cigarettes/day), and heavy smokers (larger or equal to 20 cigarettes/day) [23].

Variables related to awareness of health problems in the past 12 months were awareness of high blood pressure, diabetes, high cholesterol, heart attack or stroke, and history of alcohol use. For example, to check blood pressure history, the following question was asked: “In the past 12 months, has a doctor or health care professional told you that your blood pressure is high or that you have high blood pressure disease? “.

The response variable was considered a three-category variable with the following categories: “quit attempt and successful quit”, “quit attempt and unsuccessful quit”, and “no quit attempt and unsuccessful quit”. These categories were identified using the following questions: (1) “Have you attempted to quit smoking in the last 12 months?” and (2) “Have you stopped smoking cigarettes on a daily basis? “. If the person answered “yes” to both questions, then the person was in the “quit attempt and successful quit” category. If the person answered “yes” to the first question and “no” to the second question, then the person was in the “quit attempt and unsuccessful quit” category. If the person answered “no” to both questions, then the person was in the “no quit attempt and unsuccessful quit” category.

### Statistical methods and software

After removing missing values, descriptive statistics were used to describe the sample studied. In the univariate analysis, the relationships between quantitative and qualitative variables and the response were examined using ANOVA and chi-squared tests, respectively. The multinom function available in the nnet package of R4.0.3 software was then used to fit the multinomial logistic regression model based on the variables that were significant in the univariate analysis. It should be noted that the variables that were significant in the univariate analysis stage were considered input variables of the multinomial model. In the fitted multinomial model, the reference group for the outcome variable was considered to be “quit attempt and successful quit”, and ORs were calculated and interpreted on this basis.

## Results

Tables 1 and 2 shows some of the characteristics of the study participants according to the composition of the different quit attempt/no quit attempt and successful/unsuccessful quit groups. According to the results, of the 1293 participants (68.29%), 883 people did not attempt to quit. On the other hand, of the 410 people who attempted to quit, only 64 (15.61%) were successful. The mean (sd) age of all participants was 47.21 (13.65) years. The mean age of individuals who did not attempt to quit was significantly higher than the mean age of individuals who attempted to quit (*P*-value = 0.007). The proportion of workers who attempted to quit smoking and succeeded was higher than those who failed to quit, with a statistically significant difference observed between the response variable and job status (*p*-value = 0.012). Based on the result of Tabel 2 a higher proportion of smokers who did not attempt and failed to quit smoking (46.5%) were heavy smokers in the past, and a statistically significant difference in terms of smoking intensity was observed between this group and those who attempted to quit smoking (P- value = 0.002). A large proportion of smokers who had not attempted to quit and were unsuccessful (62.1%) had not received any recommendation from a phycisian, but more than half of those who had attempted to quit and were unsuccessful (55.8%) had received at least one recommendation from a phycisian.

**Table 1:** Demographical features and status

Quantitative features		attempt = Yes, quit = Yes (Mean ± SD)	attempt = Yes, quit = No (Mean ± SD)	attempt = No, quit = No (Mean ± SD)	Test Statistic	*p*-value
**Age (year)**		43.86 ± 13.99	45.88 ± 13.51	47.97 ± 13.63	F = 4.96	0.007*
**Qualitative features**		**Count (%)**				***p-value***
		**attempt = Yes, quit = Yes** **(** ***n*** **= 64)**	**attempt = Yes, quit = No** **(** ***n*** **= 346)**	**attempt = No, quit = No (** ***n*** ** = 883)**	**Total** **(** ***n*** **= 1293)**	
**Residence**	**Urban**	34(53.1)	244(70.5)	584(66.1)	862(66.7)	0.021*
	**Rural**	30(46.9)	102(29.5)	299(33.9)	431(33.3)	
**Marital status**	**Married**	54(84.4)	309(89.3)	796(90.1)	1159(89.6)	0.301
	**Other**	10(15.6)	37(10.7)	87(9.9)	134(10.4)	
**Employment Status**	**Employee**	4(6.3)	22(6.4)	85(9.6)	111(8.6)	0.012*
	**Worker**	14(21.9)	54(15.6)	114(12.9)	182(14.1)	
	**Self-employed**	32(50.0)	188(54.3)	478(54.1)	698(54.0)	
	**Retired**	4(6.3)	45(13.0)	111(12.6)	160(12.4)	
	**Unemployed**	5(7.8)	28(8.1)	82(9.3)	115(8.9)	
	**Other**	5(7.8)	9(2.6)	13(1.5)	27(2.1)	
**Years of education**	**Illiterate**	11(17.2)	35(10.1)	108(12.2)	154(11.9)	0.123
	**1–6 years**	14(21.9)	116(33.5)	299(33.9)	429(33.2)	
	**7–12 years**	28(43.8)	165(47.7)	396(44.8)	589(45.6)	
	**>12 yaers**	11(17.2)	30(8.7)	80(9.1)	121(9.4)	
**Income level**	**Less than 175$**	30(46.9)	158(45.7)	401(45.4)	589(45.6)	0.973
	**More than 175$**	34(53.1)	188(54.3)	482(54.6)	704(54.4)	
**Basic insurance**	**No**	9(14.1)	31(9.0)	89(10.1)	129(10.0)	0.450
	**Yes**	55(85.9)	315(91.0)	794(89.9)	1164(90.0)	

* indicates a significant difference at the 0.05 level.

**Table 2:** Characteristics related to smoking behaviors and awareness of health problems

Qualitative features		Count (%)				*p*-value
		attempt = Yes, quit = Yes (*n* = 64)	attempt = Yes, quit = No (*n* = 346)	attempt = No, quit = No (*n* = 883)	Total (*n* = 1293)	
**Smoking intensity in the past**	**Light smoker**	29(45.3)	139(40.2)	280(31.7)	448(34.6)	0.002*
	**moderate**	13(20.3)	86(24.9)	192(21.7)	291(22.5)	
	**Heavy smoker**	22(34.4)	121(35.0)	411(46.5)	554(42.8)	
**Physician recommendation**	**No**	34(53.1)	153(44.2)	548(62.1)	735(56.8)	< 0.001*
	**Yes**	30(46.9)	193(55.8)	335(37.9)	558(43.2)	
**Expose to secondhand smoke at home**	**No**	28(43.8)	163(47.1)	472(53.5)	663(51.3)	0.063
	**Yes**	36(56.3)	183(52.9)	411(46.5)	630(48.7)	
**Expose to secondhand smoke at work**	**No**	40(62.5)	189(54.6)	524(59.3)	753(58.2)	0.249
	**Yes**	24(37.5)	157(45.4)	359(40.7)	540(41.8)	
**Drinking alcohol**	**No**	45(70.3)	298(86.1)	766(86.7)	1109(85.8)	0.001*
	**Yes**	19(29.7)	48(13.9)	117(13.3)	184(14.2)	
**Heart attack**	**No**	63(98.4)	342(98.8)	862(97.6)	1267(98.0)	0.377
	**Yes**	1(1.6)	4(1.2)	21(2.4)	26(2.0)	
**Ischemic stroke**	**No**	63(98.4)	343(99.1)	876(99.2)	1282(99.1)	0.810
	**Yes**	1(1.6)	3(0.9)	7(0.8)	11(0.9)	
**Hypertension awareness**	**No**	55(85.9)	301(87.0)	772(87.4)	1128(87.2)	0.930
	**Yes**	9(14.1)	45(13.0)	111(12.6)	165(12.8)	
**Diabetes awareness**	**No**	61(95.3)	321(92.8)	818(92.6)	1200(92.8)	0.726
	**Yes**	3(4.7)	25(7.2)	65(7.4)	93(7.2)	
**cholestrol awareness**	**No**	58(90.6)	310(89.6)	811(91.8)	1179(91.2)	0.451
	**Yes**	6(9.4)	36(10.4)	72(8.2)	114(8.8)	

* indicates a significant difference at the 0.05 level.

**Fig. 1:**
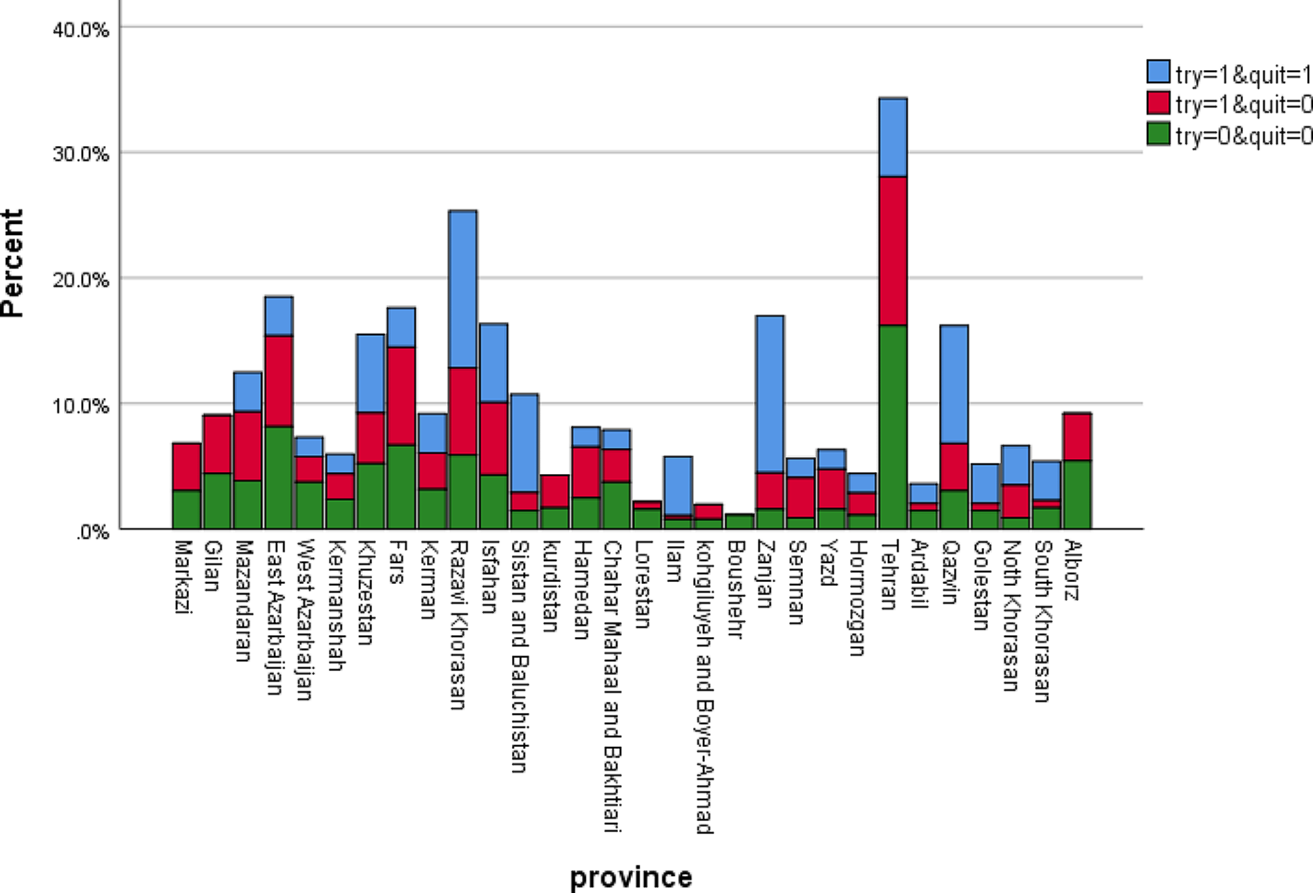
The distribution of the outcome rates among the provinces of Iran

The distribution of the outcome rates among the provinces of Iran is shown in Fig. 1. Khorasan-Razavi (12.5%), Zanjan (12.5%) and Qazvin (9.4%) had the highest rates of quit smoking.

The results of the multinomial model are presented in Table 3. This table shows the estimated coefficients of the variables, their significance, and the corresponding ORs ((OR of attempt = Yes&quit = No vs. attempt = Yes&quit = Yes) and (OR of attempt = No&quit = No vs. attempt = Yes&quit = Yes)). Compared with living in rural areas, living in urban areas increased the odds of attempting to quit and quitting unsuccessfully by more than twofold (OR = 2.192; 95% CI: 1.242, 3.871). Living in a city also increased the odds of not trying and quitting unsuccessfully by a factor of 1.71 (OR = 1.717; 95% CI: 1.002, 2.943). The odds of not trying and not quitting were almost twice as high for former heavy smokers as for light smokers (OR = 1.967; 95% CI: 1.086, 3.594). In addition, the results showed that smokers who received the phycisian recommendation were 0.599 times more likely to not attempt to quit and to not quit than smokers who did not receive the advice (OR = 0.599; 95% CI: 0.352, 1.020). In other words, the phycisian recommendation to quit smoking was a borderline significant motivating factor to quit smoking. People who drink alcohol, whether they have tried to quit or not, are 0.351 and 0.359 times more likely to quit than people who do not drink alcohol. In other words, alcohol consumption is also a protective factor against quitting smoking.

**Table 3:** Estimates and 95% confidence intervals for parameters of the multinomial model

Vriable	attempt = Yes&quit = No			attempt = No&quit = No		
	Estimate (SE)	OR (95% CI)	*P*-value	Estimate (SE)	OR (95% CI)	*P*-value
**Age (year)**	-0.007(0.012)	0.992(0.969,1.016)	0.535	0.009(0.011)	1.009(0.987,1.032)	0.423
**Residence (Ref: Rural)**						
Urban	0.785(0.290)	2.192(1.242,3.871)	0.007*	0.541(0.275)	1.717(1.002,2.943)	0.049*
**Employment Status (Ref: Employee)**						
Worker	-0.233(0.629)	0.792(0.231,2.721)	0.711	-0.889(0.594)	0.411(0.128,1.317)	0.135
Self-employed	0.247(0.584)	1.280(0.407,4.022)	0.673	-0.276(0.551)	0.758(0.257,2.238)	0.617
Retired	0.617(0.797)	1.853(0.389,8.833)	0.439	-0.033(0.764)	0.968(0.217,4.324)	0.966
Unemployed	-0.006(0.743)	0.994(0.232,4.262)	0.993	-0.524(0.703)	0.592(0.149,2.349)	0.456
Other	-0.864(0.797)	0.422(0.088,2.010)	0.278	-1.808(0.754)	0.163(0.037,0.718)	0.016*
**Smoking intensity in the past (Ref: Light smoker)**						
moderate	0.321(0.369)	1.379(0.669,2.842)	0.384	0.427(0.356)	1.533(0.763,3.082)	0.230
Heavy smoker	0.153(0.320)	1.165(0.622,2.183)	0.633	0.681(0.305)	1.967(1.086,3.594)	0.026*
**Physician recommendation (Ref: No any recommendation)**						
Yes	0.338(0.283)	1.403(0.805,2.445)	0.232	-0.513(0.272)	0.599(0.352,1.020)	0.059*
**Drinking alcohol (Ref: No)**						
Yes	-1.046(0.338)	0.351(0.181,0.682)	0.002*	-1.023(0.316)	0.359(0.193,0.668)	0.001*

* indicates a significant difference at the 0.05 level.

## Discussion

The present study is the first national survey to investigate the factors influencing smoking cessation attempts and successful smoking cessation among adult Iranian men. According to the results of this study, 31.71% of smokers made a quit attempt. On the other hand, only 15.61% of those who attempted to quit were successful. This percentage is higher than that reported in studies conducted in countries such as China (4.4%) [24] and Bangladesh (4.3%) [8].

According to the results of this study, the average age of people who attempted to quit smoking and were successful was lower than the average age of other people. This finding was consistent with the results of the study conducted by Arancini et al. [25]. This finding may be because classic theories of addiction, such as disease models [26] and learning [27], show that the more often an addictive substance is used, the stronger the addiction. This is also true for smoking, and older smokers are more addicted than younger smokers because of their longer smoking history [25]. On the other hand, although the National Institutes of Health in the United Kingdom recommends that smoking cessation programs be offered to all smokers regardless of age, their current policies target specific groups, such as pregnant women and socioeconomically disadvantaged populations, and older smokers are generally not recognized as a priority group [28].

According to the findings, people living in urban areas are less likely to quit smoking than people living in rural areas. This may be because potential stressors, such as difficulty accessing public services, high levels of crime and violence, exposure to gang activity, and increased racism and discrimination, are greater for urban residents [29, 30]. For some smokers, stress increases smoking and decreases the willingness to quit.

According to the results of Table 3, one of the factors influencing smoking cessation was alcohol consumption. Many studies in this area have documented the negative relationship between alcohol consumption and the likelihood of quitting smoking. Based on the information from these studies, alcohol consumption is associated with quitting smoking through both biological and behavioral mechanisms [31]. Biologically, alcohol consumption affects nicotine dependence and treatment success [32]. Behaviorally, smokers who consume alcohol have reported smoking more during alcohol consumption in social settings such as clubs [33]. Based on the results of Table 3, there was an inverse relationship between physician recommendation and not attempting to quit smoking, such that physician recommendation had a protective effect, i.e., people who received physician recommendation to quit smoking were more likely to attempt and successfully quit smoking. This finding is consistent with many studies [34]. For example, the results of a Cochrane review that combined data from 17 clinical trials showed that a brief recommendation from a physician significantly increased the rate of quitting smoking [35]. Therefore, focusing on this issue can be a significant public health benefit and an effective component in the development of a national tobacco control program [36].

According to the results, most of the smokers who did not attempt and failed to quit were heavy smokers in the past. This finding was consistent with the results of a study conducted in Bangladesh by Hakim et al. According to the results of this study, current smokers who smoked 10 to 19 cigarettes per day (moderate smokers) were less likely to attempt to quit smoking [8]. This finding contrasts with the study by Ni et al., in which smoking intensity was not independently predictive of quit success [37].

The strengths of the present study include the large sample size and the comprehensiveness of the study, which means that this sample is a national sample of all Iranian men.

### Limitations

The use of a self-report questionnaire can lead to some biases, such as social desirability bias and recall bias. Also, the person completing the survey may be influenced by embarrassment. Another limitation of the present study is that language or variations in cultural dialects or education level may affect the understanding of the STEPS questionnaire.

## Conclusions

According to the results of this study, heavy smokers with a history of smoking, alcohol users, and residents of urban areas were less likely to attempt and succeed in quitting smoking. By focusing on the factors that influence attempts to quit and successfully quit, strategies can be adopted by health policy makers that lead to long-term cessation and subsequently reduce smoking prevalence and related health consequences.

## Data Availability

The datasets presented in this article are not readily available because the authors need to grant permission to the NationalInstitute of Health of Iran. Requests to access the datasets should be directed to Ali-Asghar Kolahi, a.kolahi@sbmu.ac.ir.
